# Correction: Ramírez-Morros et al. Sex Differences in Cardiovascular Prevention in Type 2: Diabetes in a Real-World Practice Database. *J. Clin. Med.* 2022, *11*, 2196

**DOI:** 10.3390/jcm11154492

**Published:** 2022-08-02

**Authors:** Anna Ramírez-Morros, Josep Franch-Nadal, Jordi Real, Mònica Gratacòs, Didac Mauricio

**Affiliations:** 1 DAP-Cat Group, Unitat de Suport a la Recerca de la Catalunya Central, Institut Universitari d’Investigació en Atenció Primària Jordi Gol (IDIAP Jordi Gol), 08272 Sant Fruitós de Bages, Spain; amramirez.cc.ics@gencat.cat; 2 Gerència Territorial de la Catalunya Central, Institut Català de la Salut, 08272 Sant Fruitós de Bages, Spain; 3 DAP-Cat Group, Unitat de Suport a la Recerca de Barcelona, Institut Universitari d’Investigació en Atenció Primària Jordi Gol (IDIAP Jordi Gol), 08007 Barcelona, Spain; josepfranch@gmail.com (J.F.-N.); jreal@idiapjgol.info (J.R.); monica.gratacos@gmail.com (M.G.); 4 Center for Biomedical Research on Diabetes and Associated Metabolic Diseases (CIBERDEM), Instituto de Salud Carlos III, 08907 Barcelona, Spain; 5 Department of Endocrinology and Nutrition, Hospital de la Santa Creu i Sant Pau and Sant Pau Biomedical Research Institute (IIB Sant Pau), 08041 Barcelona, Spain; 6 Department of Medicine, University of Vic and Central University of Catalonia, 08500 Vic, Spain

## Error in Figure

In the original publication [1], there was a mistake in Figure 2. This presents smoothing line charts with changes in LDL-c, total cholesterol, and statin treatment across age in subjects on primary prevention (A) and secondary prevention (B) by gender (LDL-c, low-density lipoprotein cholesterol) as published. The mistake was that primary prevention (A) and secondary prevention (B) erroneously show the same chart. The corrected Figure 2 is as follows: Smoothing line charts with changes in LDL-c, total cholesterol, and statin treatment across age in subjects on primary prevention (A) and secondary prevention (B) by gender (LDL-c, low-density lipoprotein cholesterol). This is shown below. 

The authors apologize for any inconvenience caused and state that the scientific conclusions are unaffected. This correction was approved by the Academic Editor. The original publication has also been updated.

## Figures and Tables

**Figure 2:**
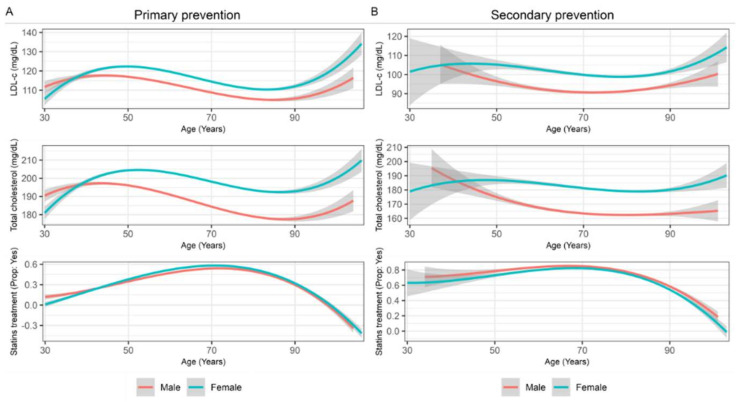
Smoothing line charts with changes in LDL-c, total cholesterol, and statin treatment across age in subjects on primary prevention (**A**) and secondary prevention (**B**) by gender (LDL-c, low-density lipoprotein cholesterol).

## References

[1] Ramírez-Morros A., Franch-Nadal J., Real J., Gratacòs M., Mauricio D. (2022). Sex Differences in Cardiovascular Prevention in Type 2: Diabetes in a Real-World Practice Database. J. Clin. Med..

