# Supplementary material for: Sex Differences in Cardiovascular Prevention in Type 2: Diabetes in a Real-World Practice Database
Source: J Clin Med. 2022 Apr 14;11(8):2196. doi: 10.3390/jcm11082196 (PMC9032335; doi:10.3390/jcm11082196)
Supplement: Supplementary file 1 [file jcm-11-02196-s001.zip › Figure S1.pdf]

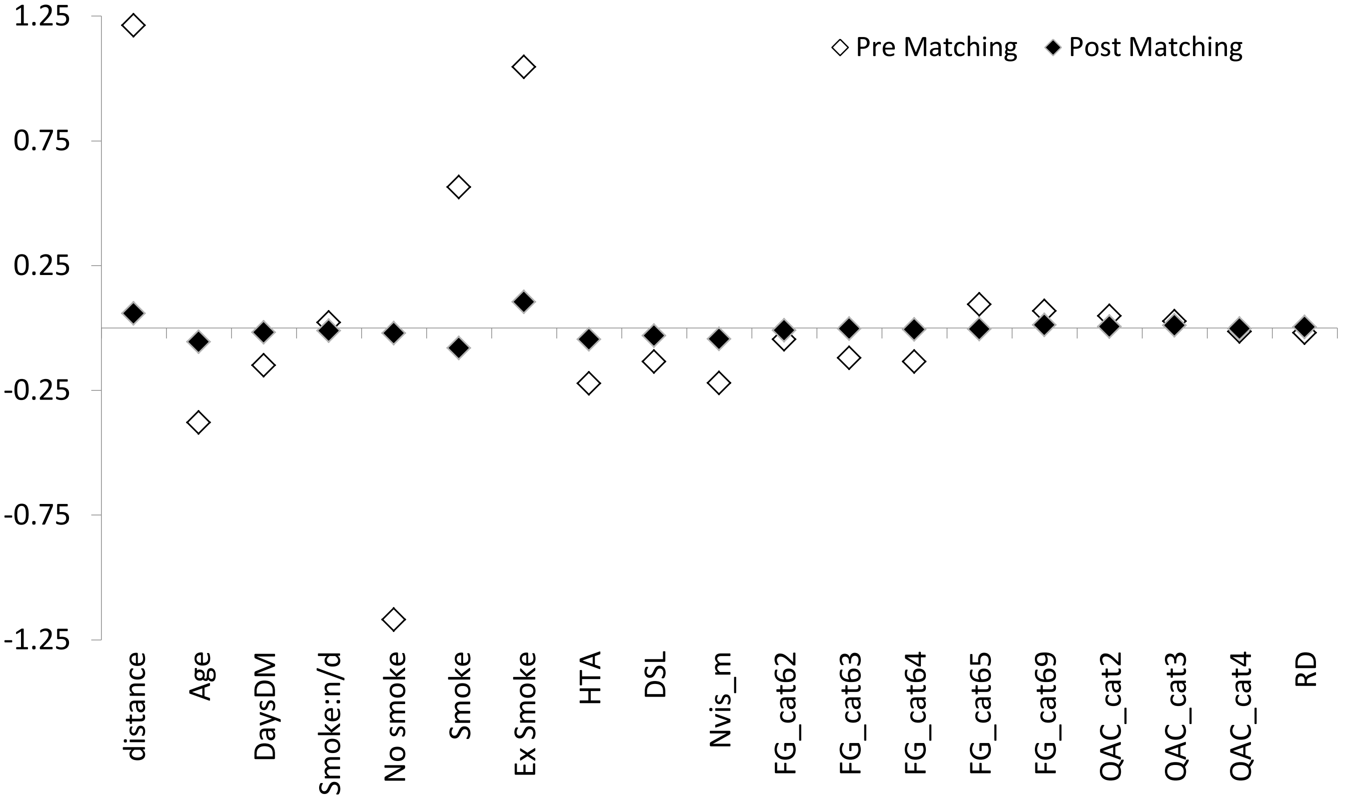

Figure S1. Absolute differences between patients with T2DM according gender in primary prevention (DaysDM, days since type 2 diabetes mellitus diagnosis; Smoke: n/d, smoking status not known; ExSmoke, former smoker; HTA, hypertension; DSL, dyslipidemia; Nvis_m, number of visits done in primary healthcare last year; FG_cat62, glomerular filtration rate <15 mL/min; FG_cat63, glomerular filtration rate 15–30 mL/min; FG_cat64, glomerular filtration rate 31–44 mL/min; FG_cat65, glomerular filtration rate 45–59 mL/min; FG_cat69, glomerular filtration rate >60 mL/min; QAC_cat2, urinary albumin creatinine ratio <30 mg/g; QAC_cat3, urinary albumin creatinine ratio 30–300 mg/g; QAC_cat4, urinary albumin creatinine ratio >300 mg/g; RD, diabetic retinopathy).
